# Comparing the Profile of Temperament and Character Dimensions in Patients with Major Depressive Disorder and Bipolar Mood Disorder with a Control Group 

**Published:** 2017-07

**Authors:** Shahram Hajirezaei, Abolfazl Mohammadi, Mehdi Soleimani, Fatemeh Rahiminezhad, Mohammad Reza Mohammadi, C. Robert Cloninger

**Affiliations:** 1 Department of Psychiatry, Tehran University of Medical Sciences, Tehran, Iran.; 2 Psychiatric and Psychology Research Center, Roozbeh Hospital, Tehran University of Medical Sciences, Tehran, Iran.; 3 Center for Psychobiology of Personality, Sansone Center for Well-Being, Washington University St. Louis, St. Louis, MO, USA.

**Keywords:** *Bipolar Mood Disorder*, *Control Group*, *Major Depressive Disorder*, *Personality Profile*, *Temperament and Character Inventory*

## Abstract

**Objective:** This study was conducted to compare the profile of temperament and character dimensions in patients with major depressive disorder and bipolar mood disorder with a control group.

**Method:** In this causal-comparative study, the population consisted of 2 clinical groups (major depressive disorder and bipolar mood disorder) and a non-clinical group. The sample was 193 individuals (77 patients with major depressive disorder, 86 patients with bipolar mood disorder, and 30 controls), with an age range of 18 to 65 years and the mean age of 40.1. They were selected from Roozbeh psychiatric hospital using available sampling method. Tools used in this research included Temperament and Character Inventory-140 and General Health Questionnaire-28. Collected data were analyzed by independent t test and one-way analysis of variance using Statistical Package for the Social Sciences-22 software.

**Results:** The results revealed a significant difference among groups in dimensions of novelty seeking, harm avoidance, persistence, self-directedness, and cooperativeness (P <0.05). The results showed that the mean was different in males and females only in the novelty seeking dimension (P <0.05).

**Conclusion:** In general, our results revealed that patients with major depressive disorder and bipolar mood disorder have different personality profiles in some dimensions of temperament and character compared with the control group.

The relationship between mental status and emotional disorders has been discussed since old medical theories about human and fundamental descriptions of Aristotle on melancholic temperament for centuries (1). Emil Kraepelin was the first person who coherently described and classified mental diseases, especially manic-depressive category of mental diseases. Manic depression is commonly associated with characteristics such as depression, restlessness, mania, or cyclothymic disorder. His works founded mainstream of clinical observations role in mental disorders (2).

A theoretical model of Kraepelin subsequently inspired many theories considering personality role in emotional disorders (3, 4). Cloninger provided a general model that included normal and abnormal personality. Moreover, he provided Temperament and Character Inventory, used broadly in patients with emotional disorders, to measure his personality theory (1, 5-7). 

According to the psychosocial model that he provided to explain temperament components, temperament systems in the brain have a functional organization with different and independent systems for activation, maintenance, and behavioral inhibition in response to certain groups of stimuli. Behavioral activation was in response to new stimuli, presence of reward symptoms, and escape of punishment. Therefore, individual differences in such capability are called novelty seeking. Behavioral inhibition was in response to punishment stimuli and lack of reward. Individual differences in behavioral inhibition are called harm avoidance. Behavior is reinforced with reward usually continues for some time after discontinuation of the reward. Cloninger called individual differences in continuation of response after discontinuation of reward as reward dependence, and persistence as individual differences in the brain system to regulate intermittent reinforcement. On the other hand, character refers to individual differences in ideas related to self-concept on the goals and values. Both temperament and character are inherited, but they have differences based on different types of learning and memory that regulate in the brain (1, 8). Three dimensions of character are as follow: self-directedness, which is the ability to adjust and adapt a behavior to respond to demands to achieve the selected goals of an individual; cooperativeness measures the extent of one’s helps and supports to others; and self-transcendence, which is the ability to remember the past and clear image of future with details for personal growth and based on the self-concept as a part of the world and its surrounding sources, associated with thoughts of mysterious presence, religious, dignity, and unconditional patience (1, 5-7).

Cloninger personality model and tools proposed to measure it, Temperament Questionnaire (TPQ) and Temperament and Character Inventory (TCI), are widely used in patients with mood disorders. Although studies conducted to compare the dimensions of temperament and character in healthy individuals and patients with mood disorders (major depressive disorder and bipolar mood disorder) have provided controversial results, almost all patients with emotional disorders obtained high scores in harm avoidance (9-13). However, it seems that bipolar mood disorder is characterized by high scores of novelty seeking (10, 12), reward dependence (11), and self-transcendence (10-13).

In the meta-analysis conducted in 2011 on the dimensions of temperament, it was confirmed that high harm avoidance was observed patients with major depressive disorder and bipolar disorder in most studies, however, with the higher score of this dimension in major depressive disorder, low scores in novelty seeking and reward dependence in patients with major depressive disorder rather than those with bipolar mood disorder (14-20). In addition, some dimensions of temperament and character may be influenced by demographic characteristics so that females obtain higher scores in harm avoidance, reward dependence, and cooperativeness, and by increase in age, scores of novelty seeking reduce and cooperativeness scores increase (21-27). When we examine personality traits, these variables can be considered as a mediating factor. However, no study has been conducted on a psychiatric sample to compare the profile of personality using the revised version of Temperament and Character Inventory in Iran. Therefore, this study was conducted to compare the profile of temperament and character dimensions of patients with major depressive disorder and bipolar mood disorder with a control group.

## Materials and Methods

***Participants***

The sample of the study consisted of 193 participants, 77 patients (40%) with major depressive disorder (males 48% vs. females 52%), 86 patients (45.5%) with bipolar mood disorder (males 40% vs. females 60%), and 30 patients (15.5%) in the control group (males 55% vs. females 45%), with the age range of 18 to 65 years. The diagnosis was performed based on psychiatric interview by psychiatrists of Roozbeh psychiatric hospital, Tehran, Iran. Patients with mood disorders were those who referred to the outpatient clinic of Roozbeh hospital in a period of 9 months from January 2015 to September 2016. Inclusion criteria for patients with mood disorders were as follow:

Having a mood disorder according to DSM-IV criteria (28); age above 18; and holding an educational level of higher than eighth grade. 

Exclusion criteria of the study were as follow: neurological disorders including dementia, epilepsy; mental retardation (IQ less than 70); and schizophrenia spectrum diagnoses. To screen the control group, General Health Questionnaire was used and 30 accompanies of patients were included in the final analysis as a control group.

***Temperament and Character Inventory-140 (TCI-140)***

TCI-140 provided by Cloninger contains 140 phrases and it is used to evaluate personality features of mentally healthy and unhealthy individuals. It includes 2 parts of temperament (including 4 dimensions of novelty seeking, reward dependence, harm-avoidance, and persistence) and Character (including 3 dimensions of self-directedness, cooperativeness, and self-transcendence), and responding to it is based on the 5-score Likert (1, 5). The previous version of this inventory (TCI) was validated and normalized in Iran by Kaviani in Tehran (29). A psychology expert who was fluent in English first translated temperament and Character Inventory-Revised (TCI-R) from English to Persian. Then, it was backtranslated to English, and the translated text was compared and confirmed with the main text by the inventor (Dr. Cloninger). It was also normalized in a study by Dabagh et al. on student population (30). Cloninger provided a shortened form of TCI-R for higher efficiency in clinical situations and named it TCI 140. Hajirezaei et al. validated TCI 140 on a sample of psychiatric outpatients in Roozbeh psychiatric hospital. The internal consistency of main dimensions, convergent validity, predictive validity, and test-retest reliability were reported to be acceptable.  ***General Health Questionnaire-28 (GHQ-28)***

General Health Questionnaire has 28 questions developed by Goldberg and Hiller and it has 4 subscales, each having 7 questions. The scales are follow: the scale of physical symptoms; the scale of anxiety symptoms and sleep disorder; the scale of social function; and the scale of depression symptoms (31). Standardization and validation of the Persian version of this questionnaire was performed by Taghavi on a sample of students in Shiraz and by Noorbala et al. in Tehran (32). In a recent study, the cutoff point of public health was 20 in the control group.

***Data analysis***

Data analysis was done using Statistical Package for the Social Sciences 22 (SPSS-22). The differences among demographic variables were determined using Chi-square test, and no significant difference was observed between demographic variables. To compare temperament and character dimensions in males and females, independent t test was used and the results are reported in Table 1. Also, to compare temperament and character dimensions among depressed patients, bipolar patients and controls, one-way analysis of variance (ANOVA) was used and to determine the differences among groups post hoc tests (LSD) were done and the results are presented in Tables 2 and 3.

## Results

Among 193 participants, 77 were male (40%) and 116 female (60%), with the mean age of 40.1 year. Marital status of the participants was as follows: 99 individuals were single (51%), 89 married (46%) and 5 divorced (3%). With respect to educational status, 25 participants did not have a high school diploma (13%), 77 had diploma (39%), 66 held a bachelor’s degree (43%), and 25 had a master’s degree and higher (13%). According to the Chi-square test results, no significant differences were found among demographic variables.

Results of one-way analysis of variance of temperament and character dimensions by means and standard deviations are demonstrated in Table 2. There were significant differences in novelty seeking, harm avoidance, persistence and self-directedness.

The mean differences between groups and significant levels are presented in Table 3. The mean differences were statistically significant in novelty seeking, harm avoidance, persistence, self-directedness and cooperativeness among groups.

The results of comparison among groups revealed significant differences among groups in dimensions of novelty seeking, harm avoidance, persistence, self-directedness, and cooperativeness (p<0.05). The results of Pearson correlation showed that novelty seeking with age (r = - 0.18, p<0.001) and cooperativeness with age (r = 0.22, p<0.001) had relatively low correlations. Moreover, novelty seeking with gender (r = -0.18, p<0.05) and cooperativeness with gender (r = 0.16, p< 0.05) indicated low correlations. On the other hand, the results of comparison of males and females demonstrated by independent t-tests revealed that just novelty seeking was statistically significant in males, with the mean of 59.79 vs. females, with mean of 56.05.

## Discussion

In general, our results showed that patients with major depressive disorder and bipolar mood disorder have different personality profile in some temperament and character dimensions compared to the control group. Therefore, it seems that by using some specific dimensions of temperament and character profile, we can develop specific case formulation and treatment plan.

**Table1:** Comparing Temperament and Character Dimensions in Males and Females

**Temperament and ** **Character Dimensions**	**Mean and Standard ** **Deviation of Males**	**Mean and Standard ** **Deviation of Females**	**Independent ** **t-tests**	**Significance ** **Level**
Novelty seeking	59.79±10.46	56.05±9.44	**2.42**	**0.016**
Harm avoidance	62.37±14.38	65.48±13.52	-1.43	0.153
Reward dependence	65.80±7.60	66.23±9.53	-0.313	0.755
Persistence	66.68±11.02	66.23±12.56	0.239	0.811
Self-directedness	58.61±15.57	56.05±14.60	1.09	0.277
Cooperativeness	70.07±10.19	72.80±10.22	-1.70	0.090
Self-transcendence	48.53±11.93	49.20±12.27	-0.352	0.725

Significant levels at p<0.05 are bolded.

**Table2:** The Summary of the Results of One-Way Analysis of Variance of Temperament and Character Dimensions to Separation of Disorder of Major Depressive Disorder and Bipolar Mood Disorder

**Temperament and ** **Character Dimensions**	**Major Depressive ** **Disorder M±SD**	**Bipolar ** **Disorder** **M±SD**	**Control ** **Group** **M±SD**	**F** **(- , -)** **(2,190)**	**Significance ** **Level**
Novelty seeking	55.01± 9.07	59.24± 9.81	55.36± 11.67	4.21	**0.016**
Harm avoidance	67.22± 13.20	62.47± 14.10	57.63± 12.75	5.95	**0.003**
Reward dependence	65.33± 8.50	66.37± 8.87	64.43± 10.85	0.589	0.556
Persistence	64.15± 10.87	68.05± 12.47	70.26± 11.62	3.76	**0.025**
Self-directedness	56.05± 14.17	57.83± 16.23	68.30± 13.36	7.46	**0.001**
Cooperativeness	72.23± 10.17	71.09± 10.53	75.80± 10.06	2.31	0.102
Self-transcendence	47.28± 11.10	50.58± 12.94	49.76± 9.29	1.65	0.194

Significant levels are bolded.

**Table3:** The Summary of the Results of Fisher's least significant difference (LSD) Post Hoc Tests

**Temperament and ** **Character Dimensions**	**Group**	**Group**	**Mean ** **Difference**	**Significant Levels**
Novelty seeking	depressed patients	bipolar Patients	- 4.23	0.007
Harm avoidance	Depressed patients Depressed patients	bipolar Patients control group	4.74 9.58	0.027 0.001
Persistence	Depressed patients Depressed patients	bipolar Patients control group	- 3.90 - 6.11	0.035 0.016
Self-directedness	Depressed patients bipolar Patients	control group control group	-12.24 -10.46	0.000 0.001
Cooperativeness	bipolar Patients	control group	-4.70	0.033

Significant levels at p<0.05 are bolded.

In previous studies, it was shown that age and gender affect some temperament and character dimensions (5, 7, 26, 27, 33-36). Our results that indicated a reverse correlation between novelty seeking and age and a positive correlation between novelty seeking and cooperativeness are in line with results of previous studies (5, 7, 13, 25, 27, 33, 35 and 37). However, unlike previous studies in which females received higher scores in harm avoidance, reward dependence, and cooperativeness (17, 21-24, 26, 27, 38, 39), in the current study, the only significant difference was in higher scores of novelty seeking by males, while no significant difference was found in other dimensions. It seems that as most of the studies were done to validate and standardize the questionnaire and were different from other studies in sample size and type, this difference was also seen in their results. The results of comparison among the 3 groups showed that patients with bipolar and depressive disorders were significantly different from the control group in self-directedness. Our results were consistent with those of previous research (9-11, 17, 18, 26, 40-45). Unlike previous studies (9, 17, 26, 42), self-transcendence was not significantly different in the 3 groups, and only depressive mood disorder was significantly different with bipolar and control group in harm avoidance, and no significant difference was found between bipolar and control groups, which was inconsistent with the result of previous study. It seems that high harm avoidance scores and low self-directedness scores in depressed patients simultaneously cause them to describe themselves with features such as fatigue, shyness, prone to worry, and pessimistic compared to the control group and bipolar disorder group. High harm avoidance generally shows susceptibility to mood disorders. Other findings showed low persistence dimension in patients with depressive and bipolar disorder compared to the control group. As shown in previous studies (20, 46, 47), low persistence was confirmed even after reducing the severity of depression in patients with mood disorders compared to controls. It seems that patients with learned helplessness are less prone to seek and achieve their goals. It also seems that weakness in decision-making skills, perseverance, and effort in these patients makes them to be more prone to helplessness and hopelessness when faced with failures and obstacles. In addition, high score of novelty seeking in bipolar patients in previous studies (10, 48, 49) has shown that high scores of novelty seeking encourages people to impulsive behaviors and experience pleasant affairs excessively, and low scores in cooperativeness in bipolar patients was also in line with previous study (20).

## Limitations

As the only way for diagnosis was psychiatric interview and archived diagnoses, and it is possible to change the diagnosis over time, it is recommended that other diagnostic tools such as Structured Clinical Interview for DSM and structured interview be used during study implementation. Although we tried to match the control group with other groups in demographic variables, full matching was not performed in the education level. It is also recommended to compare the other anxiety and obsessive-compulsive disorders with major depressive disorder. Moreover, special course of a disease in acute phase or remission of an illness should be included in the future studies.

## Conclusion

In general, our results revealed that patients with major depressive disorder and bipolar mood disorder have different personality profiles in some dimensions of temperament and character compared with the control group.

## References

[1] Cloninger CR (2004). Feeling good: the science of well-being.

[2] Von Zerssen D, Akiskal HS (1998). Personality factors in affective disorders: Historical developments and current issues with special reference to the concepts of temperament and character. J Affect Disord.

[3] Akiskal HS, Djenderedjian AH, Rosenthal RH, Khani MK (1977). Cyclothymic disorder: Validating criteria for inclusion in the bipolar affective group. Am J Psychiatry.

[4] Akiskal HS, Placidi GF, Maremmani I, Signoretta S, Liguori A, Gervasi R (1998). TEMPS-I: delineating the most discriminant traits of the cyclothymic, depressive, hyperthymic and irritable temperaments in a nonpatient population. J Affect Disord.

[5] Cloninger CR (1994). Temperament and personality. Curr Opin Neurobiol.

[6] Cloninger CR, Svrakic DM (1997). Integrative psychobiological approach to psychiatric assessment and treatment. Psychiatry.

[7] Cloninger CR, Svrakic DM, Przybeck TR (1993). A psychobiological model of temperament and character. Arch Gen Psychiatry.

[8] Cloninger CR (2009). Evolution of human brain functions: the functional structure of human consciousness. Aust N Z J Psychiatry.

[9] Engström C, Brändström S, Sigvardsson S, Cloninger R, Nylander P-O (2004). Bipolar disorder: I. Temperament and character. J Affect Disord.

[10] Nowakowska C, Strong CM, Santosa CM, Wang PW, Ketter TA (2005). Temperamental commonalities and differences in euthymic mood disorder patients, creative controls, and healthy controls. J Affect Disord.

[11] Osher Y, Cloninger CR, Belmaker RH (1996). TPQ in euthymic manic-depressive patients. J Psychiatr Res.

[12] Young LT, Bagby RM, Cooke RG, Parker JDA, Levitt AJ, Joffe RT (1995). A comparison of Tridimensional Personality Questionnaire dimensions in bipolar disorder and unipolar depression. Psychiatry Res.

[13] Zaninotto L, Souery D, Calati R, Di Nicola M, Montgomery S, Kasper S (2015). Temperament and character profiles in bipolar I, bipolar II and major depressive disorder: Impact over illness course, comorbidity pattern and psychopathological features of depression. J Affect Disord.

[14] Kampman O, Poutanen O (2011). Can onset and recovery in depression be predicted by temperament? A systematic review and meta-analysis. J Affect Disord.

[15] Bensaeed S, Ghanbari Jolfaei A, Jomehri F, Moradi A (2014). Comparison of Temperament and Character in Major Depressive Disorder Versus Bipolar II Disorder. Iran J Psychiatry Behav Sci.

[16] Jylhä P, Mantere O, Melartin T, Suominen K, Vuorilehto M, Arvilommi P (2011). Differences in temperament and character dimensions in patients with bipolar I or II or major depressive disorder and general population subjects. Psychol Med.

[17] Sasayama D, Hori H, Teraishi T, Hattori K, Ota M, Matsuo J (2011). Difference in Temperament and Character Inventory scores between depressed patients with bipolar II and unipolar major depressive disorders. J Affect Disord.

[18] Loftus ST, Garno JL, Jaeger J, Malhotra AK (2008). Temperament and character dimensions in bipolar I disorder: A comparison to healthy controls. J Psychiatr Res.

[19] Celikel FC, Kose S, Cumurcu BE, Erkorkmaz U, Sayar K, Borckardt JJ (2009). Cloninger's temperament and character dimensions of personality in patients with major depressive disorder. Compr Psychiatry.

[20] Bensaeed S, Jolfaei AG, Jomehri F, Moradi A (2014). The relationship between major depressive disorder and personality traits. Iran J Psychiatry.

[21] Fresan A, Robles-Garcia R, Lopez-Avila A, Cloninger CR (2011). Personality differences according to age and sex in a Mexican sample using the Temperament and Character Inventory-Revised. Compr Psychiatry.

[22] Farmer RF, Goldberg LR (2008). A psychometric evaluation of the revised Temperament and Character Inventory (TCI-R) and the TCI-140. Psychol Assess.

[23] Giakoumaki SG, Karagiannopoulou L, Rozsa S, Zouraraki C, Karamaouna P, Cloninger CR (2016). Psychometric properties of the Greek TCI-R and its clinical correlates: schizotypy and the self-regulation of affective and cognitive functioning. PeerJ.

[24] Goncalves DM, Cloninger CR (2010). Validation and normative studies of the Brazilian Portuguese and American versions of the Temperament and Character Inventory - Revised (TCI-R). J Affect Disord.

[25] Gutierrez-Zotes A, Labad J, Martorell L, Gaviria A, Bayon C, Vilella E (2015). The revised Temperament and Character Inventory: normative data by sex and age from a Spanish normal randomized sample. PeerJ.

[26] Hansenne M, Delhez M, Cloninger CR (2005). Psychometric properties of the temperament and character inventory-revised (TCI-R) in a Belgian sample. J Pers Assess.

[27] Pelissolo A, Mallet L, Baleyte JM, Michel G, Cloninger CR, Allilaire JF (2005). The Temperament and Character Inventory-Revised (TCI-R): psychometric characteristics of the French version. Acta Psychiatr Scand.

[28] GUZE SB (1995). Diagnostic and Statistical Manual of Mental Disorders, 4th ed. (DSM-IV). American Journal of Psychiatry.

[29] Kaviani H, Poor Naseh M (2005). Validation Of Temperament And Character Inventory (TCI) In Iranian Sample: Normative Data. Tehran University Medical Journal.

[30] Dabbagh S, CR C (2017). Validity and reliability of the Persian version of the Revised Temperament and Character Inventory -TCI-R-in Iran. J Affect Disord.

[31] Goldberg DP, Hillier VF (1979). A scaled version of the General Health Questionnaire. Psychol Med.

[32] (2009). The Validation of General Health Questionnaire- 28 as a Psychiatric Screening Tool. Hakim Health Systems Research Journal.

[33] Chen Z, Lu X, Kitamura T (2013). The factor structure of the Chinese version of the Temperament and Character Inventory: Factorial robustness and association with age and gender. Compr Psychiatry.

[34] Zaninotto L, Solmi M, Toffanin T, Veronese N, Cloninger CR, Correll CU (2016). A meta-analysis of temperament and character dimensions in patients with mood disorders: Comparison to healthy controls and unaffected siblings. J Affect Disord.

[35] Mikolajczyk E, Zietek J, Samochowiec A, Samochowiec J (2008). Personality dimensions measured using the Temperament and Character Inventory (TCI) and NEO-FFI on a Polish sample. Int J Methods Psychiatr Res.

[36] Gutierrez-Zotes JA, Cortes MJ, Valero J, Pena J, Labad A (2005). Psychometric properties of the abbreviated Spanish version of TCI-R (TCI-140) and its relationship with the Psychopathological Personality Scales (MMPI-2 PSY-5) in patients. Actas Esp Psiquiatr.

[37] Vespa A, Ottaviani M, Fossati A, Giulietti MV, Spatuzzi R, Meloni C (2015). Validation of the Italian translation of the Revised Temperament and Character Inventory--TCI-140--in adult participants and in participants with medical diseases. Compr Psychiatry.

[38] Zohar AH, Cloninger CR (2011). The psychometric properties of the TCI-140 in Hebrew. European Journal of Psychological Assessment.

[39] Martinotti G, Mandelli L, Di Nicola M, Serretti A, Fossati A, Borroni S (2008). Psychometric characteristic of the Italian version of the Temperament and Character Inventory--revised, personality, psychopathology, and attachment styles. Compr Psychiatry.

[40] Evans L, Akiskal HS, Keck Jr PE, McElroy SL, Sadovnick AD, Remick RA (2005). Familiality of temperament in bipolar disorder: support for a genetic spectrum. J Affect Disord.

[41] Farmer A, Mahmood A, Redman K, Harris T, Sadler S, McGuffin P (2003). A sib-pair study of the temperament and character inventory scales in major depression. Archives of general psychiatry.

[42] Harley JA, Wells JE, Frampton CMA, Joyce PR (2011). Bipolar Disorder and the TCI: Higher Self-Transcendence in Bipolar Disorder Compared to Major Depression. Depress Res Treat.

[43] Janowsky DS, Morter S, Hong L, Howe L (1999). Myers Briggs Type Indicator and Tridimensional Personality Questionnaire differences between bipolar patients and unipolar depressed patients. Bipolar Disorders.

[44] Nery FG, Hatch JP, Glahn DC, Nicoletti MA, Serap Monkul E, Najt P (2008). Temperament and character traits in patients with bipolar disorder and associations with comorbid alcoholism or anxiety disorders. J Psychiatr Res.

[45] Mula M, Pini S, Monteleone P, Iazzetta P, Preve M, Tortorella A (2008). Different temperament and character dimensions correlate with panic disorder comorbidity in bipolar disorder and unipolar depression. Journal of Anxiety Disorders.

[46] Matsudaira T, Kitamura T (2006). Personality traits as risk factors of depression and anxiety among Japanese students. J Clin Psychol.

[47] Peirson AR, Heuchert JW (2001). The relationship between personality and mood: comparison of the BDI and the TCI. Personality and Individual differences.

[48] Svrakic DM, Whitehead C, Przybeck TR, Cloninger CR (1993). Differential diagnosis of personality disorders by the seven-factor model of temperament and character. Archives of general psychiatry.

[49] Svrakic DM, Draganic S, Hill K, Bayon C, Przybeck T, Cloninger C (2002). Temperament, character, and personality disorders: etiologic, diagnostic, treatment issues. Acta Psychiatrica Scandinavica.

